# Poly(I:C) signaling induces robust CXCL10 production and apoptosis in human esophageal squamous cell carcinoma cells

**DOI:** 10.1007/s13577-025-01191-1

**Published:** 2025-03-03

**Authors:** Yusuke Sato, Akari Yamaya, Kento Sonoda, Akiyuki Wakita, Yushi Nagaki, Ryohei Sasamori, Yoshihiro Sasaki, Takatoshi Yoneya, Shu Nozaki, Tsukasa Takahashi, Misako Matsumoto, Tsukasa Seya, Kazuhiro Imai

**Affiliations:** 1 Department of Esophageal Surgery, Akita University Hospital, Akita, 010-8543 Japan; 2 Department of Thoracic Surgery, Akita University Graduate School of Medicine, Akita, 010-8543 Japan; 3 Nebuta Research Institute for Life Sciences, Aomori University, Aomori, 030-0943 Japan; 4 Department of Vaccine Immunology, Hokkaido University Graduate School of Medicine, Sapporo, 011-0020 Japan

**Keywords:** Toll-like receptor, TLR3, RLR, CXCL10, Esophageal squamous cell carcinoma, ESCC

## Abstract

We previously reported that high tumoral expression of Toll-like receptor 3 (TLR3) and CXCL10, a member of the CXC chemokine family, was an independent positive prognostic factor in patients with advanced thoracic esophageal squamous cell carcinoma (ESCC). However, the direct relationships between TLR3 and CXCL10 in ESCC cells was not fully understood. Here, we analyzed TLR3 mRNA and protein expression in two ESCC lines (TE8 and KYSE180) and one esophageal adenocarcinoma (EAC) line (OE19). We also assessed the effect of the TLR3 agonist poly(I:C) on production of downstream adapter proteins and cytokines, including CXCL10, and further tested its effects on cell viability and caspase 3/7 activity with and without siRNA-induced knockdown of TLR3 and the TICAM-1 or MAVS adapter protein. Both ESCC lines, but not the EAC line, showed high expression of TLR3 mRNA and protein. TICAM-1 and MAVS were also expressed, and their knockdown suppressed responsiveness to poly(I:C) in the ESCC lines. Poly(I:C) induced strong CXCL10 production, resulting in significantly upregulated caspase3/7 activity and downregulated cell proliferation in both ESCC lines but not the EAC line. The effect of poly(I:C) on CXCL10 production was attenuated after transfecting the cells with siRNAs targeting TICAM-1 or MAVS. TLR3 is thus highly expressed in ESCC cells, where it induces strong CXCL10 production and significantly upregulates caspase3/7 activity and downregulates cell proliferation. TLR3 signaling and the resultant downstream CXCL10 production have the potential to serve as useful prognostic markers and therapeutic targets for the treatment of ESCC.

**Supplementary Information:**

The online version contains supplementary material available at 10.1007/s13577-025-01191-1.

## Introduction

Squamous cell carcinoma is the predominate form of esophageal cancer in Asia, Africa and central South America and has high malignant potential [1–3]. Earlier findings suggest cigarette smoking, heavy alcohol consumption and poor oral health are all independent risk factors for upper-aerodigestive tract cancers, including esophageal squamous cell carcinoma (ESCC) [4–6]. Importantly, all of these risk factors can induce chronic inflammation [7].

Toll-like receptors (TLRs) are important immune system components that play crucial roles in innate immune responses and antigen-specific adaptive immunity against microbial pathogens [8–10]. They are classified as pattern recognition receptors (PRRs) and recognize molecular structures known as pathogen-associated molecular patterns (PAMPs), which are delivered from a wide range of pathogens, including bacteria, viruses and fungi [8, 9]. Recent evidence also indicates that in addition to PAMPs, TLRs recognize damage-associated molecular patterns (DAMPs), which are endogenous molecular patterns released from injured or dying host cells [11–13]. Persistent stimulation of TLR signaling induced by PAMPs or DAMPs sustains chronic inflammation and appears to be a potential cause of several types of cancer, including gastric cancer from Helicobacter pylori infection, hepatocellular carcinoma from hepatitis B virus (HBV) or hepatitis C virus (HCV) infection, colorectal cancer from inflammatory bowel diseases and cervical cancer from human papillomavirus (HPV) infection [14]. It is noteworthy that TLR3 and the cytoplasmic helicase MDA5, a cytoplasmic dsRNA receptor linked to the adaptor MAVS [9], sense viral dsRNA to evoke innate inflammatory responses. Within that context, it is plausible that TLR3 plays a key role in the inflammation contributing to the pathogenesis of ESCC.

Recent studies demonstrated that human TLR3 can be ectopically expressed in cancer cell endosomes [15] and that its expression levels in human ESCC cells are associated with prognosis [16]. TLR3 is expressed in myeloid cells and some epithelial cells [15, 17], but its function has also been investigated in antigen-presenting dendritic cells (DCs), particularly XCR1 + subsets, which express a high levels of TLR3 [17, 18]. XCR1 + DCs cross-prime external tumor-associated antigens to present MHC class I and facilitate antigen-specific cytotoxic T lymphocyte (CTL) proliferation [17, 18]. This DC cross-priming function is augmented in response to TLR3 agonists such as poly(I:C). Upon stimulation by a TLR3 agonist, DCs contribute to tumor cell damage induced by antigen-specific CTLs [19]. On the other hand, poly(I:C) sometimes directly induces robust cytokine release and cytotoxicity in cells expressing TLR3 [19] and work as a MDA5 ligand [9]. Consequently, the actions of TLR3 that improve prognosis in cancer patients remain unclear.

CXCL10, also known as interferon gamma-induced protein 10 (IP-10), is a member of the CXC chemokine family and is known to mediate chemotaxis, apoptosis and angiostasis and to be involved in regulating cell growth [20]. In the event of a viral infection, CXCL10 production induced via TLR3 signaling promotes proinflammatory responses [21–23]. CXCL10 also reportedly contributes to the response to tumor development in several cancers [24, 25]. In ESCC, for example, CXCL10 expression inhibits cancer cell invasion and promotes migration of immune cells to the cancer microenvironment, suggesting CXCL10 exerts an inhibitory effect on ESCC progression [26, 27]. Consistent with those results, we previously reported that high expression of TLR3 and CXCL10 within tumor tissue is an independent positive prognostic factor in patients with advanced thoracic ESCC [16, 28]. The regulatory mechanisms governing CXCL10 production via TLR3 in ESCC cells remains unclear, however.

In the present study, therefore, we analyzed TLR3 mRNA and protein expression in two ESCC lines. We also assessed the effect of the TLR3 agonist poly(I:C) on production of downstream adapter proteins and cytokines, including CXCL10, and, to a lesser extent, on cell viability and caspase 3/7 activity with and without siRNA-induced TLR3 knockdown. Our findings suggest that TLR3 signaling and downstream CXCL10 production could serve as useful prognostic markers and therapeutic targets for the treatment of ESCC.

## Materials and methods

### Cell lines

This study protocol (#1495) was approved by the Ethics Committee of Akita University Graduate School of Medicine, and all experiments, particularly those using peripheral blood leukocytes (PBLs) isolated from blood samples provided by healthy donors, were performed in accordance with the Helsinki Declaration. Two ESCC lines (TE8 and KYSE180) and one EAC line (OE19) were tested. TE8 cells were obtained from the Cell Resource Center for Biochemical Research Institute of Development, Aging, and Cancer at Tohoku University, Japan and from the RIKEN BRC Cell Bank, Japan. KYSE180 cells were from the Health Science Research Resources Bank, Osaka, Japan. OE19 cells were from the European Collection of Cell Cultures. All cell lines were characterized with HLA typing and were obtained within 6 months before experiments were started. Authentication was not done by the authors [29–31]. All cell lines were cultured in RPMI1640 (Sigma-Aldrich, St Louis, MO) supplemented with 10% (v/v) heat-inactivated fetal bovine serum (FBS, GIBCO, Grand Island, NY) and antibiotics (penicillin G/streptomycin/amphotericin B, GIBCO), and were maintained in a humidified incubator under 5% CO_2_/95% air at 37 °C.

### Reverse transcription-quantitative PCR (qPCR)

As a positive control for TLR3 mRNA expression assays, PBLs were isolated from blood samples provided by healthy donors using RBC Lysis Solution^®^ (Qiagen, Hilden, Germany) according to the manufacturer’s recommendations. mRNA expression of adaptor proteins and cytokines in PBLs and ESCC cells was then assessed with qPCR. Cells were plated to a density of 5 × 10^5^ cells/well in 6-well plates and incubated for 72 h at 37 °C in 1 mL of RPMI1640 with 10% FBS. After three washes with PBS, the cells were incubated at 37 °C for an additional 72 h in 1 mL of serum-free RPMI1640 (control) without or with 10 μg/mL poly(I:C). Total RNA was then extracted from the cells using TRIzol^®^(Invitrogen, Carlsbad, CA) or RNeasy (Qiagen). The extracted RNA was quantified and evaluated for purity using a NanoDrop 2000^®^ spectrophotometer (Thermo Fisher Scientific Inc., Waltham, MA). Reverse transcription was performed as described elsewhere [16]. Primers and probes for RT-qPCR were designed on the Roche Applied Science website at the Universal ProbeLibrary Assay Design Center. The sequences of all primer sets and the corresponding Universal Probe Library probes are listed in Supplemental Table 1.

Real-time PCR was carried out as described elsewhere [16] using Power SYBR Green PCR Master Mix (Thermo Fisher Scientific Inc.) with a StepOne Real-Time PCR System (Thermo Fisher Scientific Inc.).

### Western blot analysis

Western blot analysis was carried out using standard procedures described elsewhere [32]. Briefly, aliquots containing 10 μg of protein were subjected to 10% SDS-PAGE and transferred to polyvinylidene difluoride (PVDF) membranes (ATTO, Tokyo, Japan), which were then blocked for 1 h in 5% skim milk in Tris-buffered saline containing 0.1% Tween 20 (TBS-T). After blocking, the membranes were incubated with anti-human TLR3 antibody (1:167 dilution, Novus Biologicals, CO, USA) or with mouse monoclonal anti-β-actin antibody (1:5000 dilution, SIGMA, St. Louis, MO, USA) overnight at 4 °C. This was followed by incubation for 1 h with peroxidase-conjugated anti-mouse IgG as the secondary antibody (1:2000 dilution, DAKO, Glostrup, Denmark). Immunodetection was accomplished using an ECL Western Blotting Detection System (GE Healthcare, WI, USA).

#### ELISA

Secretion of CXCL10 from ESCC cells treated with poly(I:C) was analyzed using a specific ELISA. Briefly, cells were plated to a density of 1 × or 3 × 10^3^ cells/well in 96-well plates and incubated for 72 h in 100 μL of RPMI1640 with 10% FBS. Then after washing with PBS, the cells were incubated first for 72 h with 100 μL of small interfering RNA (siRNA) targeting TLR3 or control scrambled siRNA, which was followed by an additional 72 h in 100 μL of serum-free RPMI1640 (control) without or with 10 μg/mL poly(I:C). Thereafter, the supernatant was collected from each well and analyzed using a Quantikine Human CXCL10/IP10 ELISA kit (R&D Systems, Minneapolis, MN) or a Legend MAX Human CXCL10 (IP-10) ELISA Kit (Biolegend, San Diego, CA) according to manufactures instructions.

### Cell proliferation assay

To assess the effect of poly(I:C) on ESCC cell proliferation, cells were plated to a density of 1 × 10^3^ cells/well in 96-well plates and incubated for 24 h in 100 μL of RPMI1640 with 10% FBS. After washing with PBS, the cells were incubated for an additional 72 h in 100 μL of serum-free RPMI1640 without or with 1 μg/mL or 10 μg/mL poly(I:C) (IMGENEX, San Diego, CA). Cell numbers were then determined using a CellTiter-Glo Luminescent Cell Viability Assay kit (Promega, Madison, WI). The average of the control (without poly(I:C)) wells was defined as 100%. Each sample was analyzed in 8 wells, after which the data were expressed as the mean ± SD and compared to control.

### Caspase 3/7 assay

Cells were plated in 96-well plates and incubated as described above. After washing with PBS, the cells were incubated first for 72 h with 100 μL of TLR3 siRNA #1 or control scrambled siRNA and then for an additional 72 h in 100 μL of serum-free RPMI1640 alone (control) or with 10 μg/mL poly(I:C). Caspase 3/7 activities were then assessed using a CellTiter-Glo 3/7 Assay kit (Promega). Each sample was analyzed in 4 wells, after which the data were expressed as the mean ± SD.

### siRNA experiments

TE8 and KYSE180 cells were plated in 6-well plates at a density of 2 × 10^6^ cells/well. After allowing 24 h for the cells to attach, they were transfected with silencer select siRNA TLR3 #1 (s235, 5′—GGA UAG GUG CCU UUC GUC Att—3′), stealth RNAi siRNA TLR3 #2 (HSS110815, 5′—GCA AAC CCU GGU GGU CCC AUU UAU U—3′), silencer select pre-designed siRNA MAVS (s33178, 5′—CCA AAG UGC CUA CCA CCU Utt—3′), silencer select pre-designed siRNA TICAM-1 (s45114, 5′—GAA UCA UCA UCG GAA CAG Att – 3′) or control scrambled siRNA (Invitrogen). Briefly, 2 ml of serum-free RPMI1640 containing 18 μL of Lipofectamine RNAiMAX (Invitrogen) and 6 μL of targeted siRNA or control scrambled siRNA were added to the cells, which were then incubated for 72 h. The efficiency of the siRNA transfection was determined using RT-qPCR. For caspase 3/7 assays, only siRNA #1 was used because it interfered with TLR3 mRNA expression more effectively than siRNA #2.

### Biostatistical analysis

Statistical analyses were performed using JMP15 (SAS Institute, Cary, NC) or GraphPad Prism software (version 10.3, GraphPad Software, San Diego, CA). Comparisons were made using the Wilcoxon rank sum test, Tukey’s test and the Kruskal–Wallis test, as appropriate. Values of P < 0.05 (two-sided) were considered significant.

## Results

### Expression of TLR3 mRNA and protein in ESCC lines

**Fig. 1:**
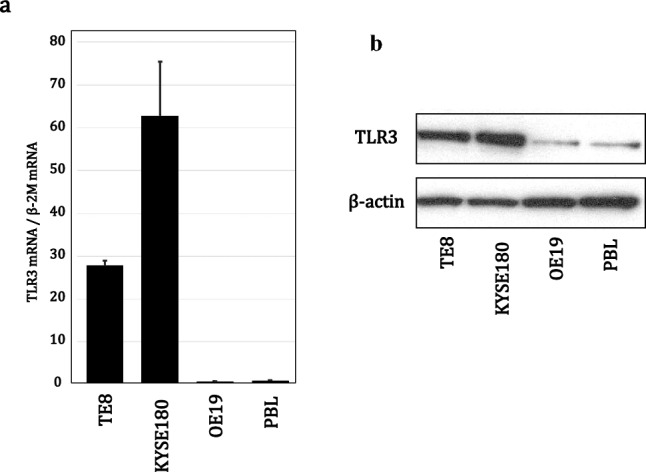
Expression levels of TLR3 mRNA and protein are elevated in ESCC cells. **a** Relative levels of TLR3 mRNA were measured in the indicated cell types with RT-qPCR. β−2 microglobulin (β−2 M) served as an internal control. The data were expressed as the mean ± SD (n = 3). The signal obtained with PBLs was assigned a value of 1. **b** Representative western blots showing levels of TLR3 protein in the indicated cell types

Using RT-qPCR, we determined the relative expression levels of TLR3 mRNA in two ESCC lines, one EAC line and PBL samples. Levels of TLR3 mRNA were about 28 times higher in TE8 cells and about 62 times higher in KYSE180 cells than in OE19 cells or PBLs (Fig. 1a). Correspondingly, western blot analysis showed expression of TLR3 protein to be markedly stronger in the ESCC cells than in EAC cells or PBLs (Fig. 1b).

### Changes in adaptor protein and cytokine mRNA production with poly(I:C) treatment

We also used RT-qPCR to assess changes in the expression of adaptor protein and cytokine mRNA in TE8 and KYSE180 cells treated with 10 μg/mL poly(I:C) (Fig. S1). Among adaptor proteins, levels of TICAM-1 and MAVS mRNAs were minimally affected by stimulation with poly(I:C), whereas NFκB2 mRNA was dramatically upregulated in both ESCC lines as compared to untreated cells. Among cytokines, IL-6, IL-8, CXCL10 and IFN-β1 mRNAs were all upregulated with poly(I:C) treatment in the two ESCC lines as compared to untreated cells. CXCL10 mRNA levels were upregulated about 74 times higher in TE8 cells and about 160 times higher in KYSE180 cells than untreated cells.

### Effect of poly(I:C) on secretion of CXCL10 protein

**Fig. 2:**
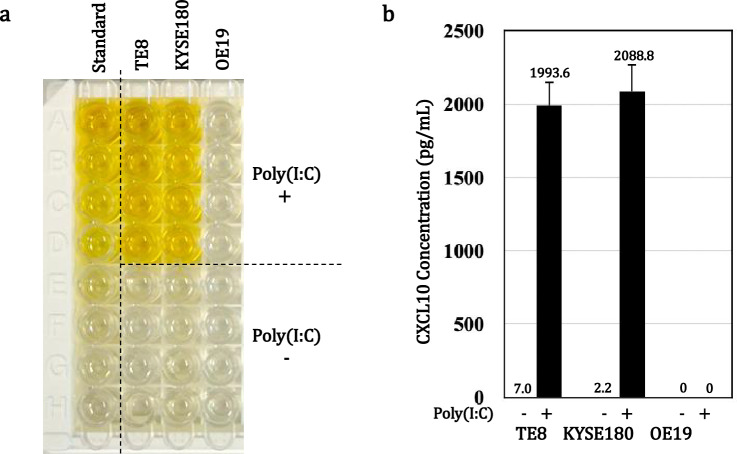
CXCL10 secretion is increased in ESCC cells treated with poly(I:C). TE8, KYSE180 and OE19 cells were treated for 72 h without or with 10 µg/mL poly(I:C) in serum-free medium, after which secreted CXCL10 was assessed using an ELISA. **a** ELISA plates. **b** Summarized measurements. The data are expressed as the mean ± SD (n = 4)

Using an ELISA, we found that after treatment with 10 μg/mL poly(I:C), CXCL10 secretion from TE8 cells into the culture medium was about 284 times higher while secretion from in KYSE180 cells was about 1044 times higher than from untreated cells (Fig. 2a). On the other hand, OE19 cells exhibited no CXCL10 secretion with or without poly(I:C) treatment (Fig. 2b).

### Effect of poly(I:C) on cell proliferation

Using cell proliferation assays, we observed that proliferation of both TE8 and KYSE180 cells was significantly downregulated after treatment with 10 μg/mL poly(I:C), and the effect was concentration dependent. On the other hand, poly(I:C) had no effect on OE19 cell proliferation (Fig. S2). The ability of poly(I:C) to suppress cancer cell proliferation was unaffected by MAVS or TICAM-1 knockdown (data not shown).

### Effect of Poly(I:C) on caspase 3/7 activity

We used caspase3/7 assays to assess apoptotic activity in cells treated with 10 μg/mL poly(I:C). Both TE8 and KYSE180 cells showed significantly upregulated caspase 3/7 activity after treatment with 10 μg/mL poly(I:C). No change in caspase 3/7 activity was detected in OE19 cells treated with the same concentration of poly(I:C) (Fig. S3).

### siRNA-induced knockdown of TLR3 mRNA expression

RT-qPCR analysis showed that two targeted siRNAs each significantly suppressed TLR3 mRNA expression in both TE8 and KYSE180 cells, as compared to untransfected control cells or cells transfected with scrambled siRNA (Fig. S4a). Moreover, we found that siRNA #1 knocked down TLR3 transcription more effectively than siRNA #2. Similarly, siRNAs targeting MAVS and TICAM-1 markedly suppressed their mRNA expression (Fig. S4b).

### Effect of poly(I:C) on CXCL10 secretion after siRNA-induced TLR3 knockdown

**Fig. 3:**
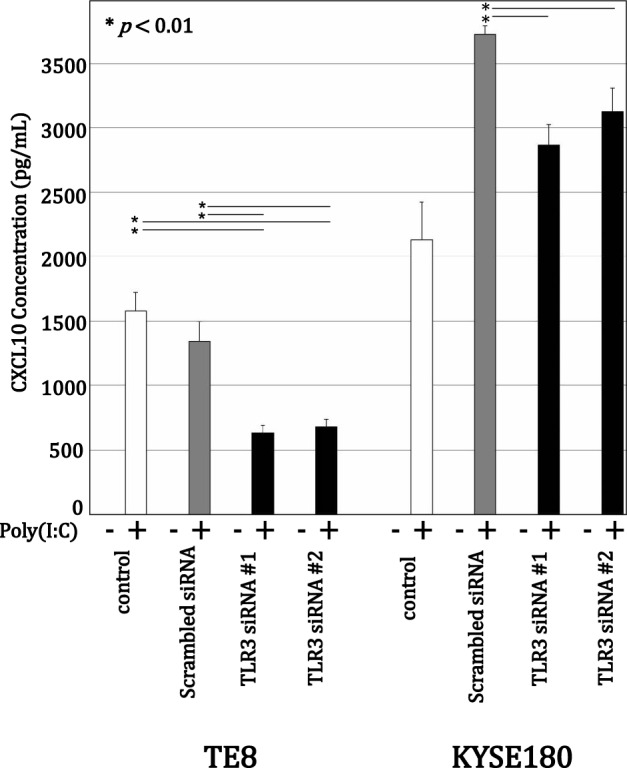
Poly(I:C)-induced CXCL10 secretion is suppressed following TLR3 knockdown. TE8 and KYSE180 cells were transfected for 72 h with TLR3 siRNA and then incubated for 72 h without or with 10 µg/mL poly(I:C) in serum-free medium. Secreted CXCL10 protein was measured using an ELISA. The data are expressed as the mean ± SD (n = 4); *p < 0.01 (Kruskal–Wallis test)

**Fig. 4:**
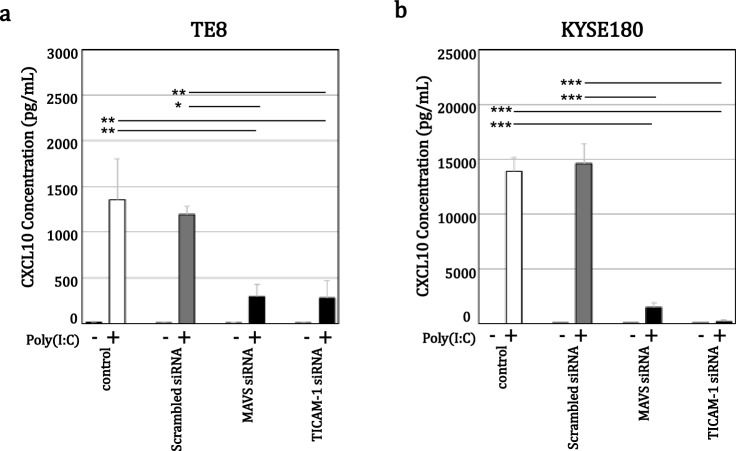
Poly(I:C)-induced CXCL10 secretion is suppressed following MAVS or TICAM-1 knockdown. TE8 and KYSE180 cells were transfected for 72 h with MAVS or TICAM-1 siRNA and then incubated for 72 h without or with 10 µg/mL poly(I:C) in serum-free medium. Secreted CXCL10 protein was measured using an ELISA. The data are expressed as the mean ± SD (n = 3); *p < 0.05, **p < 0.01, ***p < 0.001 (one-way ANOVA with Tukey test). The efficiency of MAVS or TICAM-1 knockdown was confirmed in each experiment using RT-qPCR

Following TLR3 knockdown, ELISAs showed that 10 μg/mL poly(I:C) strongly induced CXCL10 secretion (Fig. 3). In both TE8 and KYSE180 cells, transfection with TLR3 siRNA #1 or #2 significantly suppressed the poly(I:C)-induced CXCL10 secretion as compared to untreated control cells or cells transfected with scrambled siRNA. However, the impact of TLR3 knockdown was smaller than expected, which likely reflects the induction of type I IFN in by-stander cells [9]. Knocking down TICAM-1 also significantly suppressed 10 μg/mL poly(I:C)-induced CXCL10 secretion in the two ESCC lines, suggesting the TLR3/TICAM-1 pathway plays a key role in mediating CXCL10 secretion (Fig. 4). Moreover, they suggest the TLR3/TICAM-1 may contribute to previously reported CXCL10-mediated recruitment of CXCR3-positive lymphocytes to the tumor microenvironment [17–19].

### Effect of poly (I:C) on caspase 3/7 activity after TLR3 knockdown

When we assessed the effect of 10 μg/mL poly(I:C) on caspase 3/7 activity with and without TLR3 knockdown, we found that in all cases poly(I:C) treatment upregulated caspase3/7 activity as compared to untreated cells (Figs. S3 and 5). However, in both ESCC lines, transfection with siRNA #1 significantly suppressed the poly(I:C)-induced caspase3/7 activity as compared to control or scrambled siRNA. This is in contrast to the effects of MAVS or TICAM-1 knockdown, which was previously reported to have only limited effects on cell death induced by poly(I:C) in these cell lines [19].

## Discussion

In this study, we observed that two ESCC lines express TLR3 mRNA and protein significantly more strongly than an EAC line or primary PBLs. Poly(I:C), a TLR3 agonist, induced strong expression of CXCL10 mRNA and protein, which significantly enhanced caspase3/7 activity, indicating upregulation of apoptotic activity, and suppressed cell proliferation in both ESCC lines but not the EAC line. Transfection with siRNAs targeting TLR3 attenuated the effects of poly(I:C) on CXCL10 protein expression and suppressed the upregulation of caspase3/7 activity. The effects of poly(I:C) were also suppressed by TICAM-1 knockdown, indicating they are, at least in part, regulated via TLR3/TICAM-1 signaling. These observations support our earlier finding that high tumoral TLR3 and CXCL10 expression are independent positive prognostic factors in patients with advanced thoracic ESCC [16, 28].

Recent reports suggest that TLR3 signaling stimulates tumor growth in melanomas and head and neck squamous cell cancers by mediating cancer cell migration. Goto et al. reported that melanoma cell migration is enhanced after poly(I:C) [33], while Chuang et al. reported that poly(I:C) promotes migration of head and neck squamous cancer cells by stimulating secretion of IL-6 and CCL5 [34]. On the other hand, earlier reports also showed that TLR3 signaling directly or indirectly induces apoptosis in gastric cancer [35], colon cancer [36], breast cancer [37, 38], prostate cancer [39], ovarian cancer [40], hepatocellular carcinoma [41, 42] and head and neck cancer cells [43–45]. Consistent with those findings, we observed that poly(I:C) induced apoptosis in ESCC cells along with corresponding changes in CXCL10 production and caspase 3/7 activation.

It has been suggested that TLR3 ligands have three anticancer actions: (1) direct induction of apoptosis in TLR3-expressing cancer cells [19, 46], (2) activation of DC-mediated natural killer cells and CTLs [18, 47], and (3) induced secretion of chemokines, including CXCL10, from cancer cells, which recruits immune cells to the tumor microenvironment [16, 25, 48, 49]. In the present study, we observed both direct induction of apoptosis in TLR3-expressing cancer cells and strongly enhanced secretion of CXCL10 from cancer cells. Although the change in the cell death levels after MAVS or TICAM-1 knockdown was marginal, CXCL10 levels were nonetheless influenced by their knockdown, and poly(I:C) contributes to both MAVS and TICAM-1 activation [50, 51]. This suggests that poly(I:C)-induced signaling contributes to immune cell recruitment to ESCC cells via the TLR3/TICAM-1 pathway, though confirming activation of natural killer cells and recruitment of lymphocytes to the tumor microenvironment in ESCC will require further studies.

An important question is, why do ESCC cells express TLR3 so much more strongly than EAC cells or PBLs? TLR3 recognizes the dsRNA comprising the genetic material in many viruses as a specific ligand, which leads to an immunoreaction [8]. This is noteworthy, as the association between HPV and ESCC has been a topic of research, debate and conjecture for over three decades [52]. However, from their review of the literature, Ludmir et al. concluded that the notion that HPV is a prominent carcinogen in ESCC is not supported by the available results [53]. Moreover, Song et al. performed a comprehensive genomic analysis of 158 ESCC cases and found no integration of HPV, HBV or human herpes virus into the ESCC genome [54]. That said, there may still be an as-yet-unknown, and so undetected, viral infection present. In addition, DAMPs released from injured or dying host cells may induce the observed TLR3 expression in ESCC cells.

**Fig. 5:**
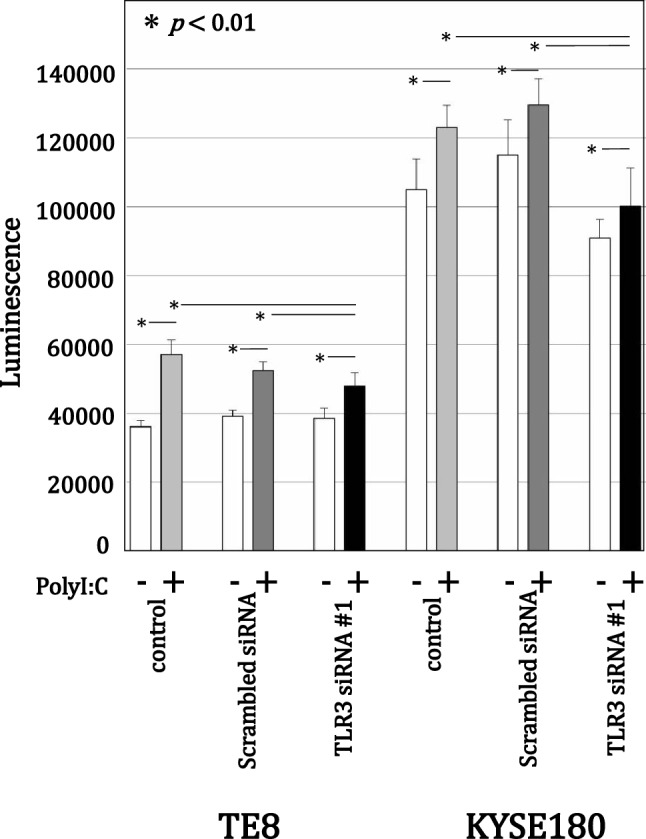
Effect of poly(I:C) on caspase 3/7 activity after TLR3 knockdown. TE8 and KYSE180 cells were transfected for 72 h with TLR3 siRNA and then incubated for 72 h without or with 10 µg/mL poly(I:C) in serum-free medium. Caspase 3/7 activity was measured using a CellTiter-Glo 3/7 Assay kit. The data are expressed as the mean ± SD (n = 4); *p < 0.01 vs. untreated cells (Kruskal–Wallis test)

An important limitation of the present study is the lack of specificity of poly(I:C). Kawai et al. reported that in addition to TLR3, poly(I:C) is recognized by several other PRRs, including retinoic acid inducible gene I (RIG-I), melanoma differentiation-associated gene 5 (MDA5) and cytoplasmic sentinels [9]. However, Luo et al. used a specific siRNA targeting TLR3 to show that poly(I:C)-induced apoptosis is mainly mediated via TLR3 in oral SCC cells [40]. Our results after MAVS knockdown suggest that either RIG-I or MDA5 is involved in the CXCL10 secretion by cancer cells and that the TLR3 pathway contributes at least as much to CXCL10 secretion as the MAVS pathway. Moreover, TICAM-1 knockdown significantly suppressed poly(I:C)-induced CXCL10 production as well as caspase 3/7 activity. Those results confirm that TLR3 is a pivotal mediator of those events in ESCCs, which is consistent with earlier findings using the TLR3-specific agonist ARNAX [18, 55]. Nonetheless, the origin of the TLR3 ligand in cancer cells remains unaddressed. Perhaps structured RNA with duplexed parts in its sequence able to mediate TLR3 activation is liberated from cancer cells [56]. Another limitation is the difficulty of assessing cell proliferation with TLR3 knockdown. Because TLR3 is an IFN-inducible gene [9], its mRNA knockdown using siRNA did not always correlate with a reduction in protein level. For this reason, we could not show clear data of cell proliferation activity with TLR3 knockdown using siRNA in Fig. S2. However, we showed that TLR3 or TICAM-1 knockdown using siRNA significantly suppressed poly(I:C)-induced CXCL10 production as well as caspase 3/7 activity in Figs. 3, 4 and 5. These data indirectly proved the inhibitory effect of poly(I:C) through TLR3 signaling on cell proliferation of ESCC cells.

In summary, we observed that TLR3 is highly expressed in ESCC lines and that its activation strongly induces expression of CXCL10, which is a major mediator of an antitumor immune response that may improve prognosis in cancer patients. These observations support our earlier findings that high tumoral TLR3 and CXCL10 expression are independent positive prognostic factors in patients with advanced thoracic ESCC. TLR3 signaling and the downstream CXCL10 production have the potential to serve as useful prognostic markers and therapeutic targets for the treatment of ESCC.

## Supplementary Information

Below is the link to the electronic supplementary material.

Supplementary file1 (DOCX 161 KB)

## Data Availability

The data that support the findings of this study are available from the corresponding author, YS, upon reasonable request.

## Abbreviations

TLRs: Toll-like receptors
CTL: Cytotoxic T lymphocyte
ESCC: Esophageal squamous cell carcinoma
EAC: Esophageal adenocarcinoma
PRRs: Pattern recognition receptors
PBL: Peripheral blood leukocyte
PAMPs: Pathogen-associated molecular patterns
Poly(I:C): Polyinosinic polycytidylic acid
RLR: Retinoic acid-inducible gene-I (RIG-I)/MDA5
MAVS: Mitochondrial antiviral signal
TICAM-1: Toll-interleukin 1 receptor domain (TIR)-containing adaptor molecule-1 (also called TRIF)
MDA5: Melanoma differentiation-associated gene 5

## References

[1] Sung H, Ferlay J, Siegel RL, et al. Global cancer statistics 2020: GLOBOCAN estimates of incidence and mortality worldwide for 36 cancers in 185 countries. CA Cancer K Clin. 2021;71:209–49. 10.3322/caac.21660 33538338

[2] Rustgi AK, El-Serag HB. Esophageal carcinoma. N Engl J Med. 2014;371:2499–509. 25539106 10.1056/NEJMra1314530

[3] Arnold M, Soerjomataram I, Ferlay J, Forman D. Global incidence of oesophageal cancer by histological subtype in 2012. Gut. 2015;64:381–7. 25320104 10.1136/gutjnl-2014-308124

[4] McCormack VA, Menya D, Munishi MO, et al. Informing etiologic research priorities for squamous cell esophageal cancer in Africa: a review of setting-specific exposures to known and putative risk factors. Int J Cancer. 2017;140:259–71. 27466161 10.1002/ijc.30292 PMC5763498

[5] Ahrens W, Pohlabeln H, Foraita R, et al. Oral health, dental care and mouthwash associated with upper aerodigestive tract cancer risk in Europe: the ARCAGE study. Oral Oncol. 2014;50(6):616–25. 24680035 10.1016/j.oraloncology.2014.03.001

[6] Baba Y, Iwatsuki M, Yoshida N, et al. Review of the gut microbiome and esophageal cancer: pathogenesis and potential clinical implications. Ann Gastroenterol Surg. 2017;1(2):99–104. 29863142 10.1002/ags3.12014 PMC5881342

[7] Hannelien V, Karel G, Jo VD, et al. The role of CXC chemokines in the transition of chronic inflammation to esophageal and gastric cancer. Biochim Biophys Acta. 2012;1825(1):117–29. 22079531 10.1016/j.bbcan.2011.10.008

[8] Akira S, Uematsu S, Takeuchi O. Pathogen recognition and innate immunity. Cell. 2006;124:783–801. 16497588 10.1016/j.cell.2006.02.015

[9] Kawai T, Akira S. Toll-like receptor and RIG-I-like receptor signaling. Ann N Y Acad Sci. 2008;1143:1–20. 19076341 10.1196/annals.1443.020

[10] Sato Y, Goto Y, Narita N, et al. Cancer cells expressing toll-like receptors and the tumor microenvironment. Cancer Microenviron. 2009;2(Suppl 1):205–14. 19685283 10.1007/s12307-009-0022-y PMC2756339

[11] Riva M, Källberg E, Björk P, et al. Induction of nuclear factor-κB responses by the S100A9 protein is Toll-like receptor-4-dependent. Immunology. 2012;137(2):172–82. 22804476 10.1111/j.1365-2567.2012.03619.x PMC3461398

[12] Wheeler DS, Chase MA, Senft AP, et al. Extracellular Hsp72, an endogenous DAMP, is released by virally infected airway epithelial cells and activates neutrophils via Toll-like receptor (TLR)-4. Respir Res. 2009;30(10):31. 10.1186/1465-9921-10-31 PMC2679007 19405961

[13] Sims GP, Rowe DC, Rietdijk ST, et al. HMGB1 and RAGE in inflammation and cancer. Annu Rev Immunol. 2010;28:367–88. 20192808 10.1146/annurev.immunol.021908.132603

[14] Ridnour LA, Cheng RY, Switzer CH, et al. Molecular pathways: toll-like receptors in the tumor microenvironment–poor prognosis or new therapeutic opportunity. Clin Cancer Res. 2013;19(6):1340–6. 23271799 10.1158/1078-0432.CCR-12-0408 PMC6314173

[15] Matsumoto M, Funami K, Tanabe M, et al. Subcellular localization of Toll-like receptor 3 in human dendritic cells. J Immunol. 2003;171(6):3154–62. 12960343 10.4049/jimmunol.171.6.3154

[16] Sato Y, Motoyama S, Wakita A, et al. TLR3 expression status predicts prognosis in patients with advanced thoracic esophageal squamous cell carcinoma after esophagectomy. Am J Surg. 2018;216(2):319–25. 29395019 10.1016/j.amjsurg.2018.01.038

[17] Bachem A, Güttler S, Hartung E, Ebstein F, Schaefer M, Tannert A, Salama A, Movassaghi K, Opitz C, Mages HW, Henn V, Kloetzel PM, Gurka S, Kroczek RA. Superior antigen cross-presentation and XCR1 expression define human CD11c+CD141+ cells as homologues of mouse CD8+ dendritic cells. J Exp Med. 2010;207(6):1273–81. 20479115 10.1084/jem.20100348 PMC2882837

[18] Matsumoto M, Tatematsu M, Nishikawa F, Azuma M, Ishii N, Morii-Sakai A, Shime H, Seya T. Defined TLR3-specific adjuvant that induces NK and CTL activation without significant cytokine production in vivo. Nat Commun. 2015;18(6):6280. 10.1038/ncomms7280. (**PMID: 25692975**). 10.1038/ncomms7280 25692975

[19] Le Naour J, Galluzzi L, Zitvogel L, Kroemer G, Vacchelli E. Trial watch: TLR3 agonists in cancer therapy. Oncoimmunology. 2020;9(1):1771143. 32934877 10.1080/2162402X.2020.1771143 PMC7466857

[20] Pradere JP, Dapito DH, Schwabe RF. The Yin and Yang of Toll-like receptors in cancer. Oncogene. 2014;33(27):3485–95. 23934186 10.1038/onc.2013.302 PMC4059777

[21] Zhang Y, Liu B, Ma Y, et al. Hantaan virus infection induces CXCL10 expression through TLR3, RIG-I, and MDA-5 pathways correlated with the disease severity. Mediators Inflamm. 2014;2014: 697837. 24701034 10.1155/2014/697837 PMC3950924

[22] Brownell J, Bruckner J, Wagoner J, et al. Direct, interferon-independent activation of the CXCL10 promoter by NF-κB and interferon regulatory factor 3 during hepatitis C virus infection. J Virol. 2014;88(3):1582–90. 24257594 10.1128/JVI.02007-13 PMC3911583

[23] Errea A, González Maciel D, Hiriart Y, et al. Intranasal administration of TLR agonists induces a discriminated local innate response along murine respiratory tract. Immunol Lett. 2015;164:33–9. 25637743 10.1016/j.imlet.2015.01.004

[24] Brownell J, Polyak SJ. Molecular pathways: hepatitis C virus, CXCL10, and the inflammatory road to liver cancer. Clin Cancer Res. 2013;19(6):1347–52. 23322900 10.1158/1078-0432.CCR-12-0928 PMC3602344

[25] Liu M, Guo S, Stiles JK. The emerging role of CXCL10 in cancer. Oncol Lett. 2011;2(4):583–9. 22848232 10.3892/ol.2011.300 PMC3406435

[26] Lu L, Pan K, Zheng HX, et al. IL-17A promotes immune cell recruitment in human esophageal cancers and the infiltrating dendritic cells represent a positive prognostic marker for patient survival. J Immunother. 2013;36(8):451–8. 23994890 10.1097/CJI.0b013e3182a802cf

[27] Yoo JY, Choi HK, Choi KC, et al. Nuclear hormone receptor corepressor promotes esophageal cancer cell invasion by transcriptional repression of interferon-γ-inducible protein 10 in a casein kinase 2-dependent manner. Mol Biol Cell. 2012;23(15):2943–54. 22675025 10.1091/mbc.E11-11-0947 PMC3408420

[28] Sato Y, Motoyama S, Nanjo H, et al. CXCL10 expression status is prognostic in patients with advanced thoracic esophageal squamous cell carcinoma. Ann Surg Oncol. 2016;23(3):936–42. 26464192 10.1245/s10434-015-4909-1

[29] Masuda M, Nishihira T, Itoh K, et al. An immunohistochemical analysis for cancer of the esophagus using monoclonal antibodies specific for modified nucleosides. Cancer. 1993;72(12):3571–8. 8252470 10.1002/1097-0142(19931215)72:12<3571::aid-cncr2820721205>3.0.co;2-9

[30] Shimada Y, Imamura M, Wagata T, et al. Characterization of 21 newly established esophageal cancer cell lines. Cancer. 1992;69(2):277–84. 1728357 10.1002/1097-0142(19920115)69:2<277::aid-cncr2820690202>3.0.co;2-c

[31] Rockett JC, Larkin K, Darnton SJ, et al. Five newly established oesophageal carcinoma cell lines: phenotypic and immunological characterization. Br J Cancer. 1997;75(2):258–63. 9010035 10.1038/bjc.1997.42 PMC2063267

[32] Wakita A, Motoyama S, Sato Y, et al. REG Iα activates c-Jun through MAPK pathways to enhance the radiosensitivity of squamous esophageal cancer cells. Tumour Biol. 2015;36(7):5249–54. 25656613 10.1007/s13277-015-3183-y

[33] Goto Y, Arigami T, Kitago M, et al. Activation of Toll-like receptors 2, 3, and 4 on human melanoma cells induces inflammatory factors. Mol Cancer Ther. 2008;7(11):3642–53. 19001446 10.1158/1535-7163.MCT-08-0582 PMC3480738

[34] Chuang HC, Huang CC, Chien CY, et al. Toll-like receptor 3-mediated tumor invasion in head and neck cancer. Oral Oncol. 2012;48(3):226–32. 22070917 10.1016/j.oraloncology.2011.10.008

[35] Qu J, Hou Z, Han Q, et al. Poly(I:C) exhibits an anti-cancer effect in human gastric adenocarcinoma cells which is dependent on RLRs. Int Immunopharmacol. 2013;17(3):814–20. 24029594 10.1016/j.intimp.2013.08.013

[36] Nojiri K, Sugimoto K, Shiraki K, et al. The expression and function of Toll-like receptors 3 and 9 in human colon carcinoma. Oncol Rep. 2013;29(5):1737–43. 23467704 10.3892/or.2013.2322

[37] Salaun B, Coste I, Rissoan MC, Lebecque SJ, et al. TLR3 can directly trigger apoptosis in human cancer cells. J Immunol. 2006;176(8):4894–901. 16585585 10.4049/jimmunol.176.8.4894

[38] Galli R, Paone A, Fabbri M, Zanesi N, et al. Toll-like receptor 3 (TLR3) activation induces microRNA-dependent reexpression of functional RARbeta and tumor regression. Proc Natl Acad Sci U S A. 2013;110(24):9812–7. 23716670 10.1073/pnas.1304610110 PMC3683754

[39] Harashima N, Inao T, Imamura R, Okano S, et al. Roles of the PI3K/Akt pathway and autophagy in TLR3 signaling-induced apoptosis and growth arrest of human prostate cancer cells. Cancer Immunol Immunother. 2012;61(5):667–76. 22038398 10.1007/s00262-011-1132-1 PMC11029084

[40] Van DN, Roberts CF, Marion JD, Lépine S, et al. Innate immune agonist, dsRNA, induces apoptosis in ovarian cancer cells and enhances the potency of cytotoxic chemotherapeutics. FASEB J. 2012;26(8):3188–98. 22532440 10.1096/fj.11-202333 PMC3405273

[41] Chew V, Chen J, Lee D, Loh E, et al. Chemokine-driven lymphocyte infiltration: an early intratumoural event determining long-term survival in resectable hepatocellular carcinoma. Gut. 2012;61(3):427–38. 21930732 10.1136/gutjnl-2011-300509 PMC3273680

[42] Chew V, Tow C, Huang C, Bard-Chapeau E, et al. Toll-like receptor 3 expressing tumor parenchyma and infiltrating natural killer cells in hepatocellular carcinoma patients. J Natl Cancer Inst. 2012;104(23):1796–807. 23197495 10.1093/jnci/djs436 PMC3814220

[43] Luo Q, Hu S, Yan M, Sun Z, et al. Activation of Toll-like receptor 3 induces apoptosis of oral squamous carcinoma cells in vitro and in vivo. Int J Biochem Cell Biol. 2012;44(8):1266–75. 22568929 10.1016/j.biocel.2012.04.025

[44] Park JH, Jeon DI, Yoon HE, Kwon SM, et al. Poly I: C inhibits cell proliferation and enhances the growth inhibitory effect of paclitaxel in oral squamous cell carcinoma. Acta Odontol Scand. 2012;70(3):241–5. 22181939 10.3109/00016357.2011.640278

[45] Rydberg C, Månsson A, Uddman R, Riesbeck K, et al. Toll-like receptor agonists induce inflammation and cell death in a model of head and neck squamous cell carcinomas. Immunology. 2009;128:e600–11. 19740321 10.1111/j.1365-2567.2008.03041.x PMC2753959

[46] Vacchelli E, Sistigu A, Yamazaki T, Vitale I, Zitvogel L, Kroemer G. Autocrine signaling of type 1 interferons in successful anticancer chemotherapy. Oncoimmunology. 2015;4(8): e988042. 26405588 10.4161/2162402X.2014.988042 PMC4570096

[47] Akazawa T, Ebihara T, Okuno M, et al. Antitumor NK activation induced by the Toll-like receptor 3-TICAM-1 (TRIF) pathway in myeloid dendritic cells. Proc Natl Acad Sci U S A. 2007;104(1):252–7. 17190817 10.1073/pnas.0605978104 PMC1765444

[48] Chew V, Abastado JP. Immunomodulation of the tumor microenvironment by Toll-like receptor-3 (TLR3) ligands. Oncoimmunology. 2013;2(4): e23493. 23734310 10.4161/onci.23493 PMC3654580

[49] Limagne E, Nuttin L, Thibaudin M, Jacquin E, Aucagne R, et al. MEK inhibition overcomes chemoimmunotherapy resistance by inducing CXCL10 in cancer cells. Cancer Cell. 2022;40(2):136–52. 35051357 10.1016/j.ccell.2021.12.009

[50] Oshiumi H, Matsumoto M, Funami K, Akazawa T, Seya T. TICAM-1, an adaptor molecule that participates in Toll-like receptor 3-mediated interferon-beta induction. Nat Immunol. 2003;4(2):161–7. 12539043 10.1038/ni886

[51] Yoneyama M, Kikuchi M, Natsukawa T, et al. The RNA helicase RIG-I has an essential function in double-stranded RNA-induced innate antiviral responses. Nat Immunol. 2004;5(7):730–7. 15208624 10.1038/ni1087

[52] Al-Haddad S, El-Zimaity H, Hafezi-Bakhtiari S, et al. Infection and esophageal cancer. Ann N Y Acad Sci. 2014;1325:187–96. 25266025 10.1111/nyas.12530

[53] Ludmir EB, Stephens SJ, Palta M, et al. Human papillomavirus tumor infection in esophageal squamous cell carcinoma. J Gastrointest Oncol. 2015;6(3):287–95. 26029456 10.3978/j.issn.2078-6891.2015.001 PMC4397257

[54] Song Y, Li L, Ou Y, et al. Identification of genomic alterations in oesophageal squamous cell cancer. Nature. 2014;509(7498):91–5. 24670651 10.1038/nature13176

[55] Miyazaki A, Yoshida S, Takeda Y, Tomaru U, Matsumoto M, Seya T. Th1 adjuvant ARNAX, in combination with radiation therapy, enhances tumor regression in mouse tumor-implant models. 2024; (submitted). 10.1016/j.imlet.2024.106947 39603425

[56] Tatematsu M, Nishikawa F, Seya T, Matsumoto M. Toll-like receptor 3 recognizes incomplete stem structures in single-stranded viral RNA. Nat Commun. 2013;4:1833. 23673618 10.1038/ncomms2857

