# Supplementary material for: Poly(I:C) signaling induces robust CXCL10 production and apoptosis in human esophageal squamous cell carcinoma cells
Source: Hum Cell. 2025 Mar 3;38(3):63. doi: 10.1007/s13577-025-01191-1 (PMC11876272; doi:10.1007/s13577-025-01191-1)

**Fig. S1**

Effect of poly(I:C) on expression of NFκB, cytokines and adaptor proteins in two ESCC lines. **a**, **b** Relative levels of NFκB, cytokines and adaptor protein mRNAs were measured using RT-qPCR in TE8 and KYSE180 lines. β-2 microglobulin or β-actin served as an internal control. The data are expressed as mean values (n = 3). The signals obtained with untreated cells were assigned a value of 1.

**Fig. S2**

Effect of poly(I:C) on cell proliferation in two ESCC lines. TE8, KYSE180 and OE19 cells were treated for 72 h without or with 1 or 10 µg/mL poly(I:C) in serum-free medium. Cell proliferation was assayed by measuring the cellular ATP levels using a CellTiter-Glo Luminescent Cell Viability Assay kit. The signal obtained with untreated cells was assigned a value of 100%. Other cell proliferation percentiles are expressed relative to the value obtained with untreated cells. The data are expressed as the mean ± SD (n = 8); **p*< 0.01 vs. untreated cells (Kruskal-Wallis test).

**Fig. S3**

Effect of poly(I:C) on caspase 3/7 activity. TE8, KYSE180 and OE19 cells were treated for 72 h without or with 10 µg/mL poly(I:C) in serum-free medium. Caspase 3/7 activities were measured using a CellTiter-Glo 3/7 Assay kit. The data are expressed as the mean ± SD (n = 4); *p* values are vs. the respective untreated control cells (Wilcoxon rank sum test).

**Fig. S4**

Efficiency of TLR3 (**a**), MAVS and TICAM-1 (**b**) knockdown in two ESCC cell lines. TE8 and KYSE180 cells were transfected for 72 h with the indicated siRNA. The efficiency of the siRNA transfection was determined using RT-qPCR. For TLR3, two siRNAs (siRNA #1 and siRNA #2) were tested. The data are expressed as the mean ± SD (n = 3); **p* < 0.05 vs. each untreated cell (Kruskal-Wallis test).


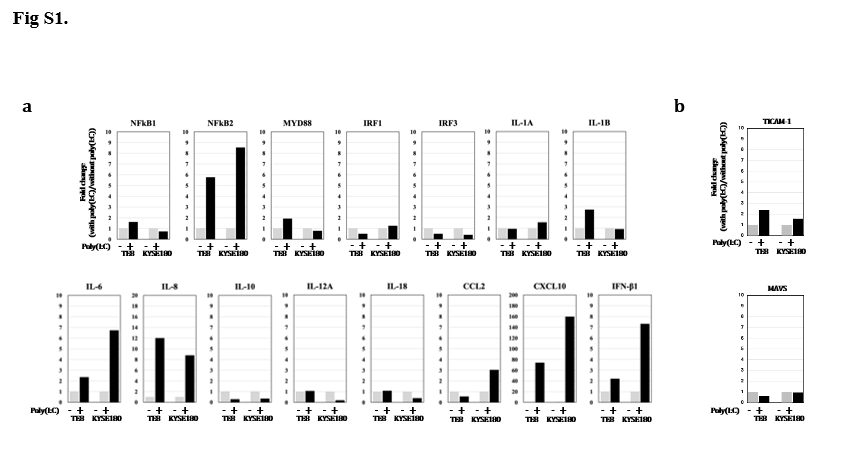


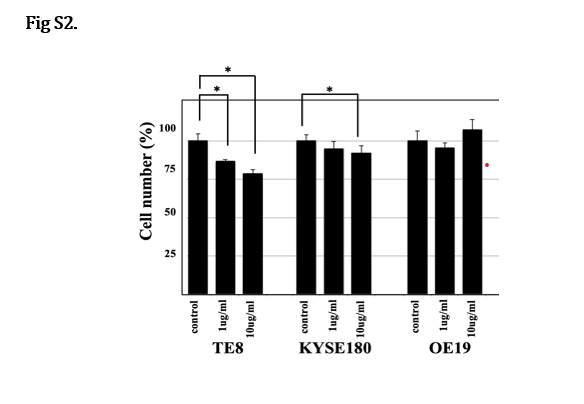

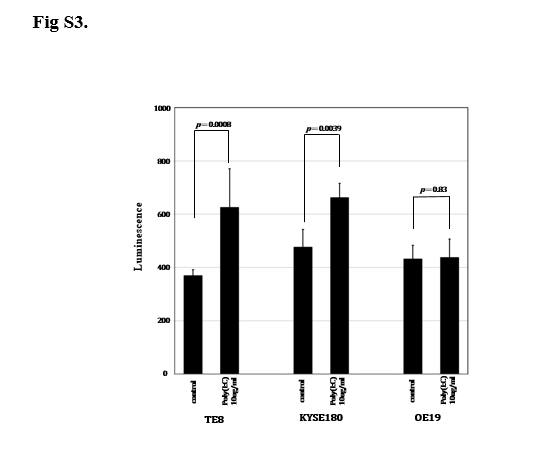


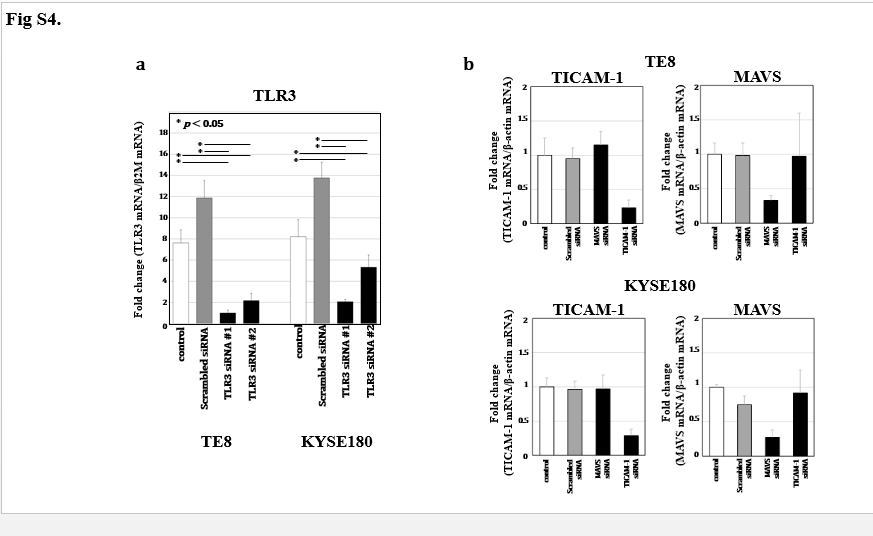

Supplement: Supplementary file 1 — Supplementary file1 (DOCX 161 KB) [file 13577_2025_1191_MOESM1_ESM.docx]
